# Security, Privacy, and Usability in Continuous Authentication: A Survey

**DOI:** 10.3390/s21175967

**Published:** 2021-09-06

**Authors:** Ahmed Fraz Baig, Sigurd Eskeland

**Affiliations:** 1 Norwegian Computing Center, 0373 Oslo, Norway; sigurd@nr.no; 2 Department of Information Security and Communication Technology, Norwegian University of Science and Technology, 2815 Gjøvik, Norway

**Keywords:** continuous authentication, security, privacy, usability, user experience

## Abstract

Continuous authentication has been proposed as a possible approach for passive and seamless user authentication, using sensor data comprising biometric, behavioral, and context-oriented characteristics. Since these are personal data being transmitted and are outside the control of the user, this approach causes privacy issues. Continuous authentication has security challenges concerning poor matching rates and susceptibility of replay attacks. The security issues are mainly poor matching rates and the problems of replay attacks. In this survey, we present an overview of continuous authentication and comprehensively discusses its different modes, and issues that these modes have related to security, privacy, and usability. A comparison of privacy-preserving approaches dealing with the privacy issues is provided, and lastly recommendations for secure, privacy-preserving, and user-friendly continuous authentication.

## 1. Introduction

We are dependent on computing technology to store and process our personal data. We interact with devices in the form of smart-phones, cars, sensors, Internet of Things (IoT), and other devices. Authentication ensures that the given entity is one it claims to be [1]. Authentication can be characterized by different factors such as knowledge-based authentication (PIN, password), possession-based (devices, smartcards, etc.), physiological-based (such as fingerprint, iris, voice, face), behavioral-based (such as keystroke dynamics, touch dynamics, motion dynamics, etc), and context-aware factors (such as physical location, IP-addresses, device-specific data, browsing history, etc.). According to a report [2], cyberattacks are happening every year, and accounts are being compromised every second. This happens due to poor implementation of authentication mechanisms. Weak passwords can be broken easily and strong passwords are not memorable. Nowadays, physiological biometric-based approaches are widely adopted in smart devices that use face recognition and fingerprints recognition, which are examples of what we refer to as authentication modes or simply “modes”. These approaches attempt to improve the usability over conventional authentication approaches. A disadvantage about physiological biometrics is that physiological features are static, which can be reproduced by an adversary.

In general, user authentication can be performed on a device or a server-side: (1) The user will authenticate himself towards the mobile device. Device-side authentication is performed entirely on the device [3]. (2) The user will authenticate himself towards a cloud service. By means of his user credentials, the user proves his authenticity to the cloud (authentication server), which performs the user authentication and grants the user access to the service if the authentication succeeded. High-performance computational resources and on-demand availability enable users and companies to leverage cloud-based services. Many mobile devices are using cloud-based services for data processing and storage, which facilitate access to multiple services and also provide easy backup to data. In a “traditional” setting, the user authenticates himself at the beginning of a session. In the case of device-side authentication, the session normally lasts as long as the user is using the device actively, and after that, it locks automatically. In the case of service/cloud-side authentication, the user provides some authentication credentials to the server, which then determines whether the user is authentic or not and on that basis grants the user access to the service.

A potential problem about session-oriented approaches is that if the user leaves the computer or the device for some time, a malicious user accessing the device in the meantime is not prevented from using it or any services that the user is logged onto. This issue could be mitigated by security mechanisms that continuously re-authenticate the user during the session. User authentication can be conducted actively or passively. The former requires explicit user attention or user action, such as entering a password or pin or putting a finger on the finger print scanner. The latter is a seamless and transparent form of authentication that runs in the background without notifying the user or requiring any user attention.

Continuous authentication (CA) offers advantages with regard to usability and security. It passively re-authenticates users without notifying the user or requiring any user attention, and it locks the system automatically in case the user is inactive or when it observes irregularities or anomalous behaviors. Continuous authentication utilizes physiological biometrics pattern recognition, behavioral biometrics pattern recognition, and context-aware authentication modes, sometimes in combination. Combining multiple modes is sometimes referred to as multi-modal authentication.

Usability pertains to ease of access, user friendliness, how satisfactorily or efficient a product or service achieves its function, and how prone it is to errors (ISO 9241-11:2018 [4]). Continuous authentication seeks to offer some trade-off between security, privacy, and usability due to its passive and seamless operation. It continuously monitors users actions and behaviors, which achieves security and usability, but not privacy as authentication mechanisms are conceptually security mechanisms. Since continuous authentication mechanisms collect personal data, such as physiological, behavioral, and context-aware user data, this causes privacy concerns.

### Survey Contributions

This survey presents an overview of continuous authentication modes with performance comparison. Specifically, we seek to answer the following questions:

1. What privacy and security challenges are there when data processing and storage is conducted in the cloud versus locally on the device?
2. How do the different CA modes score with regard to privacy (disclosure of person-specific information about behavior, location, physiological biometric characteristics, etc.) and security (how secure authentication is obtained by a mode)?
3. What techniques are preferable for mitigating the privacy and security issues of the different CA modes?
4. What behavioral, physiological, and context-aware modes, and combinations thereof, are most suited in actual CA implementations?

Furthermore, besides these questions, this survey also discusses open challenges related to usability, challenges related to ISO/ICE standards, and other challenges regarding the applicability of continuous authentication in real-time projects. The rest of the survey is arranged as follows: Section 2 discusses preliminaries; Section 3 provides a detailed overview of several modes of continuous authentication; Section 4 discusses the security and privacy risks associated with different modes of continuous authentication. The usability and other open issues are discussed in Section 5. Section 6 discusses privacy-preserving approaches and provides future recommendations. Moreover, Section 7 discusses the list of related surveys conducted for continuous authentication. Finally, the conclusion of the survey is stated in the last section.

## 2. Preliminaries

In this section, we introduce some basic authentication concepts. According to ISO/IEC 24760-1 [5], an identity is a “set of attributes related to an entity”. Continuous authentication with physiological and behavioral biometrics utilizes user-specific biometric information (referred to as templates) for user identification, whereas context-aware continuous authentication modes use context-related information for authentication. The following subsections explain authentication properties and identity management.

### 2.1. Properties of Identification

The following are properties required for identification [6]:

- Uniqueness means that each subject should have a unique identity or a set of identities.
- Universality means that the unique identity is invariant for a period of time and within a predefined scope.
- Acceptability relates to user experience, to what extent that users will accept their identities, and to how applicable or practical they are.

### 2.2. Steps for Authentication and Data Processing

Continuous authentication can be accomplished with following steps:

- Data acquisition. Raw data are acquired by various devices that contain a number of sensors (such as accelerometer, proximity sensor, camera, magnetometer, gyroscope, GPS sensors, etc.). Human behavioral information can be attained by proximity sensors and accelerometers, while gyroscope sensors detect smartphone’s rotational motion. GPS sensors collection location data.
- Feature extraction. Obtaining a set of useful features/attributes from the collected data.
- Feature selection. This phase removes irrelevant, redundant, and noisy features and selects only the most relevant features from the set of useful features.
- Classification. This phase divides the users into two classes that agree with the authentication outcome of acceptance or rejection. Various Machine Learning (ML) algorithms can be utilized for classification purposes, such as k-Nearest Neighbours (k-NN), Neural Network (NN), Support Vector Machine (SVM), Decision Tree (DT), and many others.

The literature on continuous authentication refers mostly to a few well-known ML algorithms, such as k-NN, SVM, NN, and DT. A brief introduction of these algorithms with their advantages and limitations is given in the following subsection.

#### 2.2.1. ML Algorithms

In this subsection, we provide a brief introduction of the most common ML algorithms that are used for continuous authentication.

(1) *k*-Nearest Neighbors (*k*-NN) [7]. Given *N* feature vectors (training vectors), this algorithm identifies *k* nearest neighbors of a point in a class. The working of *k*-NN relies on the distance between feature vectors. Nearest neighbors are found by using any distance calculation algorithms, such as the Euclidean distance algorithm and the Manhattan distance algorithm, over a positive integer *k*. This algorithm selects *N* points and starts calculating distances with all its neighbor points. It places a point in *N* clusters according to the nearest distance.

*k*-NN is simple and easy to implement as it does not require training steps. However, it has challenges, because it only chooses neighbors based on distance values. Moreover, *k*-NN stores entire training data in the memory, which can be a reason for slow performance on large datasets [8].

(2) Support Vector Machine (SVM) [9] is the most utilized ML algorithm. It separates data classes into two groups by drawing a hyperplane (line). This line is called the decision boundary. Any data point that lies on one side of the boundary will be classified in one class (legitimate class), and anything that lies on the other side will be classified in another class (illegitimate class).

SVM splits classes based on their distances from one data point to the other nearest data points. SVM can be classified as a linear SVM or non-linear SVM. The linear SVM segregates data with a hyperplane with a straight boundary. In contrast, the non-linear kernel does not create straight boundaries, which implies that the non-linear algorithms utilize kernels to classify non-separable data into separable data. Many continuous authentication approaches utilize SVM for classification [10,11,12,13]. SVM can perform well on small datasets; however, like *k*-NN, it cannot perform well on large and noisy datasets [14].

(3) Neural Networks (NN) [15], also known as artificial neural networks (ANN), consist of a node layer, an input layer, one or many hidden layers, and an output layer. Every node has a weight and a threshold value that is associated with connected nodes. A node is only activated when its output value is above the threshold value. Otherwise, no data are sent to the next layer of the neural network.

NN can be further classified into following types: Feed-forward Neural Network [16], Recurrent Neural Network (RNN) [17], Multilayer Perceptron (MLP) [18], Long Short Term Memory (LSTM) [19], etc. The neural network offers many advantages, such as they store information on the entire network; they can work on incomplete information and can perform multiple jobs simultaneously [20]. Moreover, each type of NN offers distinct advantages depending on applications, such as RNN performs well on image data, LSTM is suitable for time series data, and MLP has various applications in natural language processing (NLP) and speech recognition.

(4) Decision Trees (DT) [21] is a supervised ML algorithm that builds trees by continuously splitting or classifying the input data depending on certain parameters. DT consists of a root node, internal nodes (non-leaf nodes), and leaf nodes (or terminal nodes). The root node contains complete training data, the splitting process divides decision nodes into sub-nodes over a given condition, and leaf nodes or terminal nodes are the outcomes or decisions. This algorithm recursively generates new trees from the data until it reaches a stage where it cannot further classify nodes. The leaf nodes of a decision tree contain the decisions (or classifications).

#### 2.2.2. Performance

Performance indicates how accurately and securely a method achieves authentication. This is measured by means of ratios of correct acceptances (true positives, TP), correct rejections (true negatives, TN), false acceptances (false positives, FP), and false rejections (false negatives, FP) [22]:

- Accuracy is the ratio of the number of correctly matched authorized users out of all users:
  (1) $$\mathrm{Accuracy}=\frac{\mathrm{TP}+\mathrm{TN}}{\mathrm{TP}\;+\;\mathrm{FP}\;+\;\mathrm{TN}\;+\;\mathrm{FN}}$$
- False Acceptance Rate (FAR) is the likelihood of incorrectly accepting an unauthorized user. This is typically stated as the ratio of the number of incorrect acceptances divided by the number of incorrect acceptances (FP) and correct rejections (TN):
  (2) $$\mathrm{FAR}=\frac{\mathrm{FP}}{\mathrm{FP}+\mathrm{TN}}$$
- False Rejection Rate (FRR) is the likelihood of incorrectly rejecting an authorized user. This is typically stated as the ratio of the number of incorrect rejections divided by the number of incorrect rejections (FN) and correct acceptances (TP).
  (3) $$\mathrm{FRR}=\frac{\mathrm{FN}}{\mathrm{FN}+\mathrm{TP}}$$
- Equal Error Rate (ERR) is the rate at which both FAR and FRR are equal. The lower the ERR value of a biometric system, the higher the accuracy of the system.

A brief introduction to ML approaches in different modes of continuous authentication is discussed in the following sections.

## 3. Modes of Continuous Authentication

In order to define CA clearly, we mention two definitions provided in the literature. Traore [23] defines CA as *“a new generation of security mechanisms that continuously monitor user behavior and use this as basis to re-authenticate them periodically …”* Lorena et al. [24] defines CA as *“a security mechanism that monitors user actions at every point in time … during a session and determines if that user is the legitimate one.”* These definitions are a bit limited and do not cover all aspects of CA. The first definition considers only behavioral biometrics, while the second definition does not clarify whether continuous authentication is achieved actively or passively. We propose defining continuous authentication as *continuously and passively monitoring users by means of recognizing user features and actions (i.e., physiological biometrics, behavioral biometrics, or context-aware authentication modes) during a session.*

### 3.1. Physiological Biometrics

Physiological biometrics (fingerprint recognition, face recognition, and iris recognition) are among the well-known and most commonly used traditional authentication modes. These modes are also utilized for continuous authentication.

#### 3.1.1. Face and Voice as Biometrics

Face recognition and iris recognition can be utilized for continuous authentication. A face recognition-based biometric authentication method was presented in [10]. The authors utilized the support vector machine (SVM) for experiments and recruited 32 applicants to test the prototype. Their method achieved 3.92–7.92% EER. In 2015, Crouse et al. [11] also proposed a face recognition-based continuous authentication method for mobile devices. This method collected face images of 10 applicants and trained SVM classification algorithm for experiments. They achieved 0.1–1% FAR, 73% TAR, and 64% accuracy.

Voice recognition can be used for continuous authentication. Feng et al. [25] propose a voice recognition method for continuous authentication. It was evaluated by means of 18 users. It achieved 97% recognition accuracy with 0.1% FPR. A list of studies on face-based and voice-based continuous authentication methods with performance comparison is shown in Table 1.

#### 3.1.2. ECG and EEG Features as Biometrics

Electroencephalography (EEG) measures the electrical activities of the brain signals, and electrocardiography (ECG) measures the timing and strength of heart signals. EEG and ECG are considered as unique features that can be used for user authentication [32]. Table 2 presents experimental results and a performance comparison of a few studies [33,34,35,36,37], which utilized EEG and ECG as modes for continuous authentication. Some studies combine two or more different modes (multimodal biometric) for continuous authentication such as face and fingerprint, face, iris, and voice, EEG, gait and fingerprint, EEG, eye blink, etc. Table 3 indicates performance of some multimodal biometric systems found in the literature.

### 3.2. Behavioral Biometrics

User behavior recognition can be utilized for user authentication. The following modes of behavioral biometrics are used for continuous authentication.

#### 3.2.1. Motion Dynamics

Motion dynamics are indicated by the patterns of a person’s gait or walking style. Gait-based recognition techniques can identify and differentiate human activities based on walking style. Motion dynamics data are collected from sensors, such as gyroscopes or accelerometers that are attached to the human body for data collection. Derawi et al. [52] used a Google mobile device (G1) containing embedded sensors for data collection. In this study, 51 volunteers participated in the data collection process by carrying mobile phones that had a motion sensor on the right-hand side of the hip. Hence, this method used Dynamic Time Warping (DTW) for matching and achieved 20% EER. Mäntyjärvi et al. [53] placed a sensor on the waist. They performed experiments with 36 participants and utilized FFT for matching. The proposed method achieved an accuracy of 72–88% with 7% EER. Gafurov et al. [54] attached sensors on 100 participants, whereof 30 users had sensors on the ankle, 30 users on their arms, 100 users had sensors on the hips, and 50 participants had a mobile device in their pockets. Authors utilized kNN for classification and achieved equal error rates (5%, 10%, 13%, and 7.3%), respectively. Table 4 presents a comparison of few recent gait-based recognition approaches with respect to their performance [55,56,57,58,59,60,61].

#### 3.2.2. Touch Dynamics

Touch dynamics are commonly used authentication methods for smart devices, where touch screens are used as a source for data collection. User authentication is performed by analyzing user behaviors such as gestures, swipes, or tapping on the screen. Sae-Bae et al. [62] presented a multi-touch gesture-based authentication approach by using five-finger touch gestures and movements that was tested on 34 participants. The authors utilized the Dynamic Time Warping (DTW) algorithm. Their proposed method achieved an accuracy of 90% with an ERR of 2–5%. Rauen et al. [63] utilized gesture-related data to verify users. Their method monitors different gesture activities such as how users deal with screen (pressing a button and scrolling styles). They tested their method with a random forest (RF) classification algorithm and achieved an accuracy between 99.68 and 96.26% with 3.15% FAR and 9.13% FRR. Some other studies also used touch dynamics for continuous authentication [64,65,66,67,68,69,70,71]. A performance comparison for these approaches is presented in Table 5.

#### 3.2.3. Stylometry Dynamics

Every user writes text in a unique style. Stylometric-oriented recognition techniques analyze written texts to identify a user’s identity. This mode uses sentence structure and semantics to authenticates users. Brocardo et al. [75] presented user authentication approach that verifies users by their stylometry. They divided texts into several blocks, and extracted features vectors from each block. Basic features are extracted by a combination of lexical words and lexical characters, whereas advanced features are extracted by N-gram analysis. They used the support vector machine (SVM) algorithm on two different datasets (Enron, Twitter), and achieved 9.98–21.45% EER. Kaur et al. [76] conducted experiments to recognize and analyze specific text activity by written text. They analyzed 3057 tweets with different ML algorithms (SVM, k-NN, RF, MLP). Among these 3057 tweets, their approach identified 94.38% accurately. A performance analysis of a few more studies is discussed in Table 6.

#### 3.2.4. Keystroke Dynamics

Several researchers have proposed keystroke pattern recognition for user authentication. Such techniques analyze individual typing styles on the keyboard based on the assumption that individuals handle keyboards uniquely. By registering keypress events and time duration, patterns of key latency and key-hold time can be obtained. Assuming that users have unique keystroke patterns, this can be considered as a behavioral biometrics mode and be used for recognizing users for continuous authentication.

Joyce et al. [80] introduced user authentication using keystrokes dynamics in 1990. Their proposed method measured and analyzed typing speed. Their experiments were performed on 33 participants who were asked to type a paragraph as a text. Their experiments achieved 0.25% FAR and 16.36% FRR. Gascon et al. [81] proposed a keystroke-based continuous authentication technique, where 300 participants typed short sentences on the smartphone. Typing events were recorded to analyze typing motion of the user’s fingers. They utilized SVM for matching, and their method achieved 92% TPR and 1% FPR.

The performance of the research works in [81,82,83,84] are presented in Table 7.

#### 3.2.5. Eye Movement

User behavioral features related to eye movements or eye blinks can also be utilized for continuous authentication. In 2004, Kasprowski et al. [87] introduced user authentication based on eye movements. They captured eye fixation on the object (middle and on eight edges) with the help of eye-tracking equipment. The experimental results prove that these features are useful for user authentication. Song et al. [41] captured the subjects’ focus on the screen and recorded the eye movements. Twenty participants were engaged in experiments; the proposed system achieved 88.73% accuracy with 10.61% EER. Further experimental results of a few recent studies [42,43,44] using eye-movement for continuous authentication are discussed in Table 2. Recently, Saied et al. [45] proposed an eye-blink-based user authentication system that captures eye-blink patterns and compares them during the authentication phase. They achieved an accuracy of 98.4%, which has been proven on CEW dataset [88]. Experimental results of other studies are presented in Table 8.

### 3.3. Context-Aware Authentication

Context-aware modes utilize IP-address, devices, operating systems, and other profiling parameters, such as GPS, battery usage, network usage, web browsing behaviors, and online activities to authenticate a user continuously. Yazji et al. [89] proposed an implicit authentication method by observing user activity patterns to distinguish between normal and abnormal behaviors. Their authentication method monitors user activities, such as the physical location where the files are accessed, which operations are performed on the file, the time when they access the network, and IP addresses of the source and destination. The authors performed experiments on eight users. Their authentication method achieved 90% accuracy with 13.7% FAR and 11% FRR. Gomi et al. [90] proposed browsing-based user recognition for continuous authentication. They collected and analyzed the browsing histories (in conjunction with IP addresses, URLs, and access times) of 1000 users using Linear Regression (LR) to verify the users. The authors achieved 85% accuracy with 0.03% EER. Recently, Mahbub et al. [91] utilized user app-usage patterns for continuous authentication. Their method analyzes the time and duration spent on certain applications by a specific user. Based on their analysis, they used hidden Markov models (HMMs) on two datasets (UMDAA-02, Securacy). The performance comparison for these studies is presented in Table 9.

## 4. Security and Privacy Concerns

This section discusses the criteria for secure and privacy-preserving methods by considering privacy principles [96]. Moreover, this section also discusses security and privacy issues associated with different modes of CA and possible security vulnerabilities in machine learning (ML) algorithms.

Referring to Question 2, this section discusses how do the different CA modalities score with regard to privacy and security. Privacy issues that are relevant for this paper pertain to the disclosure of person-specific information about behavior, physiological biometric characteristics, and context-aware information, such as location, etc. Security issues relevant for this paper pertain to authentication in the sense of how well the addressed authentication modalities perform. We do not consider software-related security issues, such as software vulnerabilities, nor communication security, etc.

### 4.1. Continuous Authentication Cases

Continuous authentication can be utilized to protect smart devices, such as smartphones, and also cloud-based services. In both scenarios, data can be processed either in the cloud or in the smart device.

*Case 1*. Authentication processing is performed in the cloud for the purpose of users accessing a cloud-based service. In this case, the device collects data, and continuous data processing and authentication are performed in the cloud. Hence, device processing is reduced, but considerable communication is required, which is consequently power-consuming. Importantly, this case has privacy issues due to the transmission and revealing of personal data to the cloud.

*Case 2a*. Authentication processing is performed in the device for the purpose of authenticating the user to the device. So, in this case, the device collects and processes data. The processing requires considerable memory and computational resources, which is power-consuming.

*Case 2b*. Authentication processing is done in the cloud for the purpose of authenticating the user to the mobile device. In this case, the authentication processing is outsourced to a third-party server, which, similarly with Case 1, requires considerable communication. There are, therefore, privacy issues due to the transmission and revealing of personal data to the cloud.

### 4.2. Threat Actor Assumptions

Section 4.1 sketched cases or scenarios for continuous authentication. In this section, we describe relevant threat actor assumptions for these cases.

For Case 1, we assume that there exists a curious (a.k.a. semi-malicious) insider at the server-side who wants to know about the user-specific authentication information, such as location data, IP address, or other online activities, that will be continuously transmitted from the device to the server. We also assume that the curious insider has either partial or full knowledge of training data (i.e., the template) used for physiological or behavioral biometrics, the features computation process, and feature selection criteria. The curious adversary has the capability of template reconstruction. The security issue is that Case 1 relies on the performance of authentication mode. If the adopted authentication mode does not provide good accuracy (i.e., specific mode produces high FAR), then we assume there is a threat of a masquerade attack.

**Figure 1:**
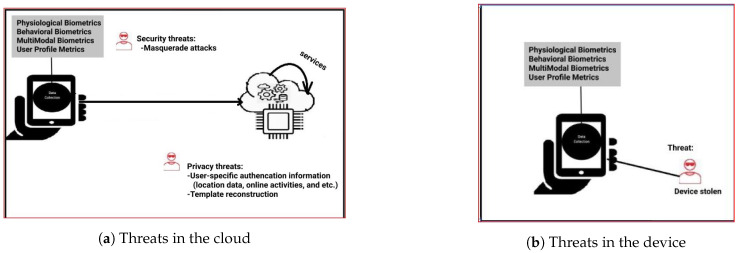
The threat model.

Considering Case 2a in Section 4.1, we assume that the mobile devices securely store user data so that an adversary with access to mobile devices cannot attain the stored templates. The security threat pertains to the performance of the modalities. This is, for instance, of relevance in case a device is stolen, as indicated in Figure 1b.

Considering Case 2b, we assume similar privacy and security threats as mentioned in Case 1, which implies that we have a threat actor that is a malicious or curious insider who has access to authentication data.

### 4.3. Security Concerns

The security of continuous authentication modalities is determined by different factors, including the performance such as accuracy, false acceptance rate (FAR), and false rejection rate (FRR) of a specific mode. Secondly, how easy is it to forge a biometric modality, and numbers of possible attacks such as mimicry attacks, template leaking attacks, cross-comparison attacks, etc. Physiological and behavioral biometric authentication mechanisms do not provide 100 percent accuracy, meaning that there are chances of false matches. In general, physiological biometric methods have better accuracy than both behavioral biometrics and context-aware authentication modes. An important point that is often overseen is that these methods are subject to certain kinds of attacks commonly referred to as replay attacks, which, in this context, could be forging fingerprints, etc. [97,98,99,100,101,102]. Moreover, physiological biometrics need segmentation, which requires more preprocessing. Behavioral biometric-based approaches, such as touch dynamics or keystroke dynamics, can be more efficient because they require less preprocessing compared to physiological biometrics. There are still arguments about whether continuous authentication modes are secure or not. These modes do not provide very good accuracy as these approaches produce a high false acceptance rate (FAR) and false rejection rate (FRR). Due to these reasons, there are possibilities of false acceptance. Moreover, continuous authentication modes with behavioral biometrics are tested on small datasets. These approaches need to be tested on more than one dataset to determine whether these modes produce the same performance, such as same accuracy, same FAR, and FRR on different datasets.

### 4.4. Privacy Concerns

For processing data in the cloud, data are outsourced to the third-part authentication server, which opens security and privacy concerns, i.e., users are not aware of what type of data is collected and stored, how these data will be used in the future, and who has access to their personal data.

Continuous authentication with different modes faces various privacy challenges. Context-aware CA modes monitor user location data obtained by GPS, online user activities, IP address, app-usage, etc. Since these data contain users’ personal information, for instance, GPS data reveals the current location of the user. Such techniques cannot protect the privacy of the user’s identity and location [103,104,105].

Continuous authentication by monitoring online activities, such as cookies or online activities, with browsing history data may disclose information about user (such as gender, age, and preferred sites) [106,107,108]. Researchers [109] performed experiments to identify users by matching anonymous browsing histories with the publicly available dataset (twitter). They achieved more than 70% accuracy; even browsing history data was in an anonymized form.

Physiological biometric templates compromise the privacy of user identity information, health information, and other biological information [110]. For instance, CA using face recognition systems to collect and store facial features, which may disclose user emotional states by analyzing facial expressions [111]. Behavioral biometric modalities can also compromise user privacy in similar ways. Behavioral biometric modalities authenticate the user by recognizing their daily life routine data, such as gait recognition, stylometry, touch dynamics, etc., which reveal current user activities. Moreover, keystroke dynamics can be used to identify user age, gender, and the hand used for typing [112]. Compromised profiles based on behavioral biometrics may reveal user identities and behavior that cannot be permanently changed like a password [113].

### 4.5. Security and Privacy Challenges in Machine Learning Algorithms

Machine learning (ML) has several applications in different fields; ML requires continuous collection of high-quality, unprecedented data. These data are uploaded to a centralized location. ML algorithms extract patterns from these data and build models, and models are updated with newly collected data [114]. Physiological and behavioral biometric-based approaches utilize ML algorithms. However, an investigative study [115] provides experimental evidence that ML approaches are vulnerable to sample inference attacks, reconstruction attacks (single and multi-sample), and label distribution estimation attacks (single and multi-sample). A study in [116] performed experiments to prove that ML models are also vulnerable to membership attacks. Moreover, another study [117] also provides common privacy breaches and attacks, such as model inversion, data de-anonymization, and model extraction attacks [118].

Machine learning models for classification, such as Support Vector Machine (SVM), k-Nearest Neighbors (kNN), and Hidden Markov Models (HMM), are mostly utilized for various continuous authentication modes. Authors [113] claim that these models (SVM and kNN) store actual user samples in users’ authentication profiles. Based on the available data, they utilized positive samples (belong to one user) and presented reconstruction attacks on mobile-based continuous authentication in the cloud, which successfully identifies users from data samples.

### 4.6. Attacks on Different Modes of Continuous Authentication

From a security point of view, continuous authentication with various modes faces different challenges. Countermeasures against various attacks on physiological biometrics have been discussed for decades [119,120], but, still, physiological biometrics are not considered secure authentication modes. Behavioral biometric-based approaches also face distinct security vulnerabilities. Touch dynamics cannot withstand adversarial generative attacks; these attacks manipulate training models to produce erroneous outcomes. Study [121] provides experimental evidence that these attacks on touch dynamics can increase EER ranging from 5% to 50%. Such attacks on keystroke dynamics can increase EER from 28% to 84% [122]. Moreover, Khan et al. [123] demonstrated in experiments on smartphones that keystroke dynamics cannot resist mimicry attacks. Kumar et al. [124] designed imitation attacks on a gait-based authentication system by imitating user gait patterns by using a digital treadmill. Classification results prove that these attacks can increase FAR from 5.8% to 43.66%. Karimian et al. [125] demonstrated the presentation attack in experiments that if an attacker captures a short template of ECG data by any means (malicious insider), these template data can be used to map attacker ECG data into the victim’s ECG data. They collected ECG templates of 52 users from Physikalisch-Technische Bundesanstalt (PTB) database for experiments. Their attacks achieved average success rates of 90% to 96%.

## 5. Usability and Other Issues

Considering the usability perspective, this section discusses practical challenges associated with the adaptation of continuous authentication (CA). In the context of usability, almost all biometric modalities face distinct challenges [126].

### 5.1. Modality-Specific Issues

Regarding Research Question 4, this section discusses the limitations of each modality in different scenarios. In real-life scenarios, the employed authentication modality needs to be determined by the user situation, i.e., what the user is doing (or not doing) at the moment. To the best of our knowledge, none of the single approaches could be suitable for all user situations.

Continuous authentication with physiological biometric-based modalities faces various challenges, such as fingerprint recognition, which requires the user to perform an action (scan fingerprint after some time). Considering the definition for continuous authentication in Section 3, fingerprint recognition conflicts with the concept of continuous authentication because it requires user attention and user action and does not authenticate users passively. Similarly, the voice recognition authentication mode does not fit well with the concept of continuous authentication, as this consequently does not work with quiet users but, in contrast, requires continuous speaking, which is not practical. Moreover, face and iris recognition modes could be utilized for continuous authentication, assuming that the user is holding the device in front of their face. Nevertheless, continuous monitoring with a camera could also affect user acceptance.

Motion-based continuous authentication basically takes the walking style (gait) into account. This implies that in cases of running or jogging, users will not necessarily be recognized or that the recognition accuracy will be lowered.

Some context-aware modes utilize only GPS data to authenticate a user continuously. These approaches are not efficacious when devices are stolen inside a specific area and, in this regard, cannot differentiate whether the user is legitimate or not. Moreover, access is denied to legitimate users when they move out of specified locations. Continuous authentication mode based on online search histories and browsing data does not provide technical details, such as how authentication will work in real scenarios, as continuous authentication requires continuous data. However, it is still unclear how the model will be trained with new data and if users search sites other than their regular routine, how they will be authenticated. Thus, these modes cannot deal with such scenarios, and due to these reasons, these modes are considered weak modes of continuous authentication.

### 5.2. Reduced Recognition Accuracy

User recognition accuracy is important for authentication security in the sense that low accuracy leads to poor authentication security. Likewise, low recognition accuracy in the sense of false rejections affect usability and will be perceived as poor usability and poor user experience [127]. Some behavioral modalities may produce high false acceptance rates (FAR) and false rejection rates (FRR), which consequently will lead to reduced security and usability.

### 5.3. Emotional States

In regard to the previous subsection, a user deals differently with a keyboard or touchscreen during stress compared to their normal mood. Emotional states (such as stress, happiness) will be a factor that also has an impact on recognition accuracy and, therefore, the usability for touch dynamic modalities and behavioral modalities in general.

### 5.4. Lack of Standards and Protocols

A list of standards has been proposed by the international electrotechnical commission (IEC) and the international standard organization (ISO). Usability follows ISO standard 9241-11 [4]; cryptographic authentication protocols follow different ISO standards: entity authentication follows ISO/IEC 9798-3 [128], message authentication using shared key follows ISO 16609:2012 [129], and zero-knowledge proofs and techniques follow ISO/IEC 9798-5 [130], while cybersecurity, information security, and privacy protection follow ISO/IEC JTC 1/SC27 [131].

However, we could not find such standards for continuous authentication. It is needed to be standardized, for instance, what estimated time a behavioral biometric-based approach could take to observe user behaviors during the enrollment phase. If the estimated time in the enrollment phase for user behavior observation is too short, then it cannot completely identify a user, which could compromise security. If the estimated enrollment time is too long, then it could affect the usability. The purpose of continuous authentication is to detect imposters immediately after the session begins. The enrollment phase of continuous authentication is different than static authentication; continuous authentication requires more time to observe user behaviors during the enrollment phase [132]. Moreover, the minimum-maximum time to block a device in case of illegitimate access and mechanisms to unblock the device also need to be standardized. Finally, it is also imperative to differentiate that continuous authentication modes, such as behavioral biometrics and context-aware modes, can be used as an identity, or these modes are only utilized for user verification. In general, behavioral biometrics and context-aware modes cannot be used solely as authentication factors; however, these modes could be used as an additional factor with ID/password.

### 5.5. Power Consumption Issue

Continuous authentication actively monitors user actions. Sensors play an essential role, especially continuous authentication with behavioral biometrics. From the data collection phase to authentication and authorization, all processes require sensors and continuous data processing [133]. The deployment and utilization of a certain amount of sensors to improve the recognition accuracy of specific activity, but it requires additional expenses of computation resources and energy consumption. Battery consumption is one of the paramount issues in a smartphone. Smartphones use a number of sensors (e.g., proximity sensors, light sensor, gyroscope, barometer, accelerometer, and a digital compass) [134]. These sensors consume a large amount of battery power. Sensory data are collected at higher power costs [135]. Few studies provide the detailed analysis on smartphone power consumption [136,137,138,139]. In general scenarios, power management could be attained by cutting off sensors’ power when they are not in use, but continuous authentication requires continuous monitoring and continuous processing, as well as the sensory power that needs to be turned on during the entire active sessions.

## 6. Recommendations for Future Research Directions

This section discusses privacy-preserving approaches and provides recommendations to propose secure and privacy-preserving methods for continuous authentication. Moreover, this section also provides recommendations to improve usability.

### 6.1. Privacy-Preserving Approaches

Continuous authentication with different modes outsources personal data to the server for authentication purposes. Compromised user accounts/profiles can cause identity theft and can also reveal user identity and other related information. These data require secure and privacy-preserving storage and processing. This section discusses privacy-preserving approaches and alludes a few recommendations to achieve secure and privacy-preserving continuous authentication.

#### 6.1.1. Cancelable Biometrics

Cancelable biometrics was introduced to solve security and privacy concerns for biometrics. Cancelable biometric approaches provide template non-reversible and biometric salting, which can increase the security and privacy of templates. Images are transformed in a way that makes it difficult to reconstruct the original image from the distorted image. Cancelable Biometrics also provides the capabilities to enroll and revoke new biometric samples, i.e., revoke the previous templates and reissue new templates in case previous templates get compromised. Few studies utilized cancelable biometrics techniques for template protection, such as the authors in [140] utilized random projection approach with the cancelable feature. Authors in the study [141] utilized the fingerprint mixing (mixing two fingerprints) technique. Moreover, a study [142] used a BioHashing interpretation-based cancelable biometric approach to enable privacy.

#### 6.1.2. Bloom Filters

A bloom filter [143] is a space-efficient probabilistic data structure of support membership queries. Bloom filters are used to determine whether a given element is a member of a set or not [144]. Bloom filters have intrinsic characteristics that offer advantages, such as the space-efficient, controlled false positive, constant-time query, etc. In recent decades, authors applied bloom filters for biometric templates. The authors in [145] used cancelable biometrics with bloom filters. Moreover, a study [146] used adaptive bloom filters for BTP. Furthermore, the authors in the study [147] also utilized bloom filters to achieve unlinkable and irreversible biometric templates.

#### 6.1.3. Homomorphic Encryption

Homomorphic encryption (HE) allows computation on encrypted data so that the data remain confidential during processing. Partially homomorphic encryption (PHE) supports either addition or multiplication at a time. In comparison, fully homomorphic encryption (FHE) supports both operations (addition and multiplication). Thus, by utilizing these homomorphic encryption techniques, the users do not need to trust the server. The users send encrypted data to the server for processing, and the server performs computation without data decryption of the data [148]. During the authentication for services, personal user data are transmitted to an (untrusted) cloud authentication service. Thus, by using homomorphic encryption (HE), we can accomplish data confidentiality. Homomorphic encryption is utilized in a few studies where the biometric data were outsourced to the server. The following studies utilized homomorphic encryption for privacy-preserving biometric authentication: [149,150,151,152,153,154,155].

#### 6.1.4. Secure Two-Party Computation

In secret sharing schemes, parties share a secret among a group of participants so that no individual can reconstruct the secret from the information available to him. Secret sharing methods enable multiple parties to cooperate with each other and construct/reconstruct the secrets. Secret sharing could be helpful with two-party computation [156] if users do not trust the cloud and do not want to outsource personal data due to privacy concerns.

In the case of the biometric authentication process, users hold their biometric samples, and biometric templates are stored in a database at the server-side. A protocol is executed to determine the similarity or dissimilarity between templates. Secure two-party computation can enable the identification without disclosing biometric data to each other. Thus, utilizing secure two-party computation and dividing the data processing resources between the client and server will be useful to achieve privacy and trust. A list of references that utilized secure two-party computation to achieve privacy: [157,158,159,160].

#### 6.1.5. Zero-Knowledge Proofs

Zero-Knowledge Proof (ZKP) [161] is considered a privacy-enhancing technique. ZKP enables secure data sharing and ensures that one party can prove itself without disclosing particular or personal information. ZKP does not allow the server to read or write user authentication data, metadata, or cryptographic keys. This technique ensures that user authentication data will remain confidential from malicious or curious insiders and external attackers even if the server gets compromised. The authors in [162] present privacy-preserving authentication with zero-knowledge proofs.

#### 6.1.6. Comparison

Many of the privacy-preserving techniques have been utilized for biometric template protection to solve security and privacy issues. However, these techniques still face distinct challenges. Privacy-preserving methods based on cryptobiometrics, such as fuzzy commitment schemes [163] and fuzzy vault schemes [164], have been utilized for biometric data protection. However, these solutions face issues related to data distinguishability and data reversibility, which cannot provide full privacy [158]. Regarding the performance of cancelable biometric approaches, these approaches can cause two problems: (1) they can obscure the feature of local neighborhoods element, and (2) during the compression phase, alignment cannot be appropriately performed [165].

For privacy-preserving context-aware modes, several statistical privacy techniques, such as *k*-anonymity [166], *l*-Diversity [167], and *t*-closeness [168] can be applied to achieve privacy-preserving continuous authentication. These techniques anonymize user identity attributes, quasi-identifiers, and other sensitive attributes that can reveal the user’s identity to achieve privacy. These techniques can also be applied to context-aware data that continuous authentication modes utilize. However, we could not find references related to the application of these approaches in continuous authentication. Experiments can be performed to see what level of privacy and accuracy an authentication system achieves by applying these statistical techniques.

Classical cryptographic approaches demand decryption before comparison, i.e., template comparison cannot be performed in the encrypted domain, implying that templates need to be decrypted during the authentication process. Decryption before authentication can enable an adversary to observe biometric templates and launch an authentication attempt. Homomorphic encryption solves the issue of decryption before authentication [169] because HE allows computation on encrypted data.

Regarding Question 3 in Section 1, and by considering the ISO standard for biometric information protection ISO/IEC 24745 [170], the security and privacy issues for continuous authentication can be mitigated by utilizing cryptographic techniques, such as homomorphic encryption with secure two-party computation and Zero-Knowledge Proofs (ZKP). However, while designing FHE, the degree of polynomials is increased by the addition of noise, which can be result in poor performance. Therefore, FHE requires applying boot-striping for noise removal. Furthermore, bloom filters also seem to be promising techniques to protect biometric information with efficient security and performance. Bloom filters can also be utilized with homomorphic encryption [171]. Continuous authentication modes suffer from significant security and privacy challenges; thus, the utilization of homomorphic encryption combined with bloom filters can solve both security and privacy challenges. Moreover, these techniques can be applied to all modes of continuous authentication, as discussed in Section 3.

### 6.2. Recommendations to Improve Usability

The usability-related issues stated in Section 5 can be improved in different ways. One aspect is to improve the usability with the help of psychology. This includes studying cognitive and social factors, such as user emotions, user behavior, and user habits, to determine the differences in users’ emotional states, such as users’ behaviors during happiness or anger. This knowledge can be utilized to design new solutions based on users’ psychological states that could improve user acceptance and usability. The other way is to ask users’ opinions by conducting a survey to know user experiences with different modes of continuous authentication.

Regarding the modality-specific issues discussed in Section 5.1 and in order for continuous authentication to be usable, a set of modalities needs to be considered that could automatically choose the authentication mode according to the scenario. Most of the literature, in general, addresses one or two modalities isolated from other modalities, i.e., these approaches are suitable for only one or two specific situations. Recently, the proposed studies [172,173] utilized multiple modalities and evaluated their approaches on different modes. Further work in this direction combined with privacy-preserving approaches can solve modality-specific and privacy issues.

## 7. Related Surveys

This section presents a brief discussion on recently published surveys on continuous authentication (CA), as shown in Table 10. In 2015, the authors presented a survey [174] focusing on a short overview of multi-biometric authentication and discussed the applicability and adoption of implicit authentication with multi-biometric authentication traits. In 2016, Patel et al. [126] presented current progress and future challenges of CA on mobile devices. Ayeswary et al. [175] also presented a brief overview of different CA methods, their merits, and demerits. Moreover, the authors explained open problems and emerging necessities of a continuous authentication system as well. Gonzalez-Manzan et al. [24] presented a comprehensive overview of different components of continuous authentication for the Internet of Things (IoT). Furthermore, this survey also focuses on the industrial status, ongoing research project contributions on continuous authentication, an overview of related standards, and different aspects proposal for future research directions for CA also presented in this survey.

In 2020, Abuhamad et al. [176] presented sensor-based behavioral biometrics, a new survey. This survey describes different behavioral biometric-based approaches and their adoption for CA on smartphones. Rasnayak et al. [177] analyzed continuous authentication from the perspective of usability and resource consumption. In addition, they prepared questionnaires in their survey and asked users’ opinions. They conducted a survey involving 500 participants. Furthermore, they showed in their conclusion that users want to utilize continuous authentication, but they want less resource-consuming methods. Furthermore, users have privacy concerns regarding their data that have been utilized for continuous authentication. Eglitis et al. [178] investigated how sensory data are collected and utilized in experiments for behavioral biometrics. Moreover, they examined 32 papers and assessed their citations and how training is performed. Recently, in 2021, the authors of [179] discussed privacy issues associated with sensor-based behavioral biometics. Moreover, they discussed a short overview of behavioral biometric-based approaches. Furthermore, they also presented the review of different available datasets, and finally, the authors also suggested recommendations that could be proven as a considerable privacy-preserving treatment for continuous authentication.

## 8. Conclusions

Continuous authentication is slightly different from static authentication. It requires efficient performance in terms of accuracy and high computation. Behavioral biometrics could be the best mode of continuous authentication due to its seamless nature. However, unfortunately, this mode does not achieve very high accuracy yet. Furthermore, other modes of continuous authentication cannot be considered strong modes due to their limitations. Moreover, we cannot ignore other issues related to usability and user experiences before applying continuous authentication in a specific domain. The aspects of security, privacy, and usability in continuous authentication require researchers and industrial attention.

In this survey, we have discussed physiological, behavioral biometrics, and context-aware modes relevant to continuous authentication. We have gathered and compared the results of different studies pertaining to continuous authentication in terms of security, privacy, and usability. Most continuous authentication modes achieve usability to some extent, but security and privacy are still questionable, in which we have identified some security and privacy risks of relevant modes. Moreover, issues related to usability, such as power consumption and lack of standards and protocols, are also identified in this survey. Finally, we have discussed privacy-preserving methods and have provided a comparison and future directions to improve security, privacy, and usability. The recommended improvements can make continuous authentication more applicable in different domains of real-world applications.

## Figures and Tables

**Table 1:** Face and voice.

Studies	Modality	Classification Algorithms	# Users	Performance
[10]	Face	SVM	32	3.92–7.92% EER
[11]	Face	SVM	10	0.1–1% FAR, 73% TAR, 64% accuracy
[26]	Face	LBP	12	82% accuracy on small-size image, 96% on 80 × 80 pixels
[12]	Face	SVM	dataset	13–30% EER
[13]	Face	SVM	dataset	94% accuracy, 0.92% TNR
[27]	Face	CNN	YouTube	0.86% EER
[25]	Voice	SVM	18	97% accuracy, 0.1% FPR
[28]	Voice	SVM	27	93% accuracy, 3% FRR
[29]	Voice	HMM	21	99% accuracy, 1% EER, 1% FRR
[30]	Voice	DTW	15	88% accuracy, 15% FRR, 0.01% FRR
[31]	Voice	HMM	12	93.3% accuracy, 1.01% EER

**Table 2:** EEG-, ECG-, and eye-movement-based authentication.

Studies	Techniques	Classification Algorithms	# Users	Performance
[33]	EEG	FFT	23	11% EER
[34]	EEG	FFT	23	79% accuracy
[35]	EEG	kNN	50	97% CRR
[38]	ECG	1DMRLBP	-	10.10% EER, 1.57% FAR, 0.39% FRR
[39]	ECG	ZMCP	19	100% accuracy, 0.36% EER
[40]	ECG	kNN-DDM	-	84.8% accuracy, 0.2% EER
[41]	Eye movement	SVM	20	88.73% accuracy, 10.61% EER
[42]	Eye movement	SRC	30	93.1% accuracy, 6.9% EER
[43]	Eye movement	SVM	22	3.93% EER
[44]	Eye movement	SVM	-	97.95% accuracy
[45]	Eye blink	CNN	CEW	98.4% accuracy
[46]	BioAura	SVM, AB	-	1.9% EER, 7.6% FAR, 9.6–8.4% FRR

**Table 3:** MultiModal biometrics.

Studies	Modality	Classification Algorithms	# Users	Performance
[36]	EEG, gait	SVM, RNN	6	63.16% FRR with EEG, 1.9% FRR with multiple modes
[37]	EEG, ECG	Euclidean	526	22.97–29.36% ERR with EEG, 0.928–8.216% ERR with multiple modes
[47]	Face, fingerprint	HMM	11	0.9995% accuracy with fingerprint, 0.970% accuracy with face
[48]	EEG, fingerprint	NBM	40	4.16% ERR with EEG, 1.12% ERR with fingerprint
[49]	Face and voice	LBP, VAD	152	HTER: 11.9% (male), 13.3% (female), EER: 10.9% (male), 10.5% (female)
[50]	EEG, eye blink	LS	31	0.89–1.1% ERR, 6.71% FAR with EEG, 2.71% FAR with multi-mode, 8.49% FRR with EEG, 2.09% FRR with multi-mode
[51]	EEG, face	BT	6	90% accuracy

**Table 4:** Gait-based authentication.

Studies	Classification Algorithm	# Users	Performance
[52]	DTW	51	20% EER
[53]	FFT	36	72–88% accuracy, 7% EER
[54]	kNN	100	85.7% accuracy, 5% EER
[55]	SVM	14	92.7% accuracy
[56]	SVM	51	53% accuracy, 33.3% EER
[57]	CNN	4007	91% accuracy, 33.3% EER
[59]	CC	15	95% accuracy, 5.5% EER
[58]	CRM	48	53% accuracy, 21.7% EER
[60]	GDI	744	66.3% accuracy, 5.6% EER
[61]	GDI	51	37% accuracy, 7.22% EER

**Table 5:** Touch dynamics.

Studies	Classification Algorithms	# Users	Performance
[62]	DTW	34	90% accuracy, 2–5% EER
[63]	RF	-	99.68–96.26% accuracy, 3.15% FAR, 9.13% FRR
[64]	DT, RF	41	12.5% FAR, 1.63% FRR
[65]	L1 distance	78	77% accuracy, 6.33–15.40% EER
[66]	(1NN), DTW	23	90% accuracy
[67]	MHD	104	92.65–93.96% accuracy, 1.55–0.31% EER
[68]	SVM		95% accuracy
[69]	SVM	40	88.5% accuracy, 5.17% FRR
[70]	PSO-RBFN	48	60% accuracy, 2.22% FAR, 2.54% FRR, 2.4% EER
[71]	MLP	20	95.96% accuracy, 6.94% FAR, 2.55% FRR
	SVM	20	94.4% accuracy, 3.7% FAR, 3.5% FRR
	LR	20	84.3% accuracy, 13.7% FAR, 14.6% FRR
	NB	20	86.7% accuracy, 14.2% FAR, 11.5% FRR
[72]	SVM	41	0–4% EER
[73]	DTW	48	77% accuracy, 21% FAR, 19% FRR
[74]	RF, J48 tree	40	4.66% FAR, 0.13% FRR

**Table 6:** Stylometry.

Studies	Classification Algorithms	# Users	Performance
[75]	SVM	datasets	9.98–21.45% EER
[76]	SVM	tweets	94.38% accuracy
[77]	SVM	76	12.42% EER
[78]	KNN	dataset	91% accuracy, 3.3% EER
[79]	SVM	67	0.004% FAR, 0.01% FRR

**Table 7:** Keystrokes Dynamics.

Studies	Classification Algorithms	# Users	Performance
[80]	-	33	0.25% FAR, 16.36% FRR
[81]	SVM	300	92% TPR, 1% FPR
[82]	kNN	20	0.08% EER
[83]	SVM	24	0.44–3.93% EER
[84]	kNN	20	97.90% accuracy, 5.1% EER
[85]	kNN	63	83.22–92.14 % Accuracy
[86]	Statistical	100	5.73% FAR, 7.27% FRR, 6.9% EER

**Table 8:** Eye-movement-based authentication.

Studies	Techniques	Classification Algorithms	# Users	Performance
[41]	Eye movement	SVM	20	88.73% accuracy, 10.61% EER
[42]	Eye movement	SRC	30	93.1% accuracy, 6.9% EER
[43]	Eye movement	SVM	22	3.93% EER
[44]	Eye movement	SVM	-	97.95% accuracy
[45]	Eye blink	CNN	CEW	98.4% accuracy
[46]	BioAura	SVM, AB	-	1.9% EER, 7.6% FAR, 9.6–8.4% FRR

**Table 9:** Context-aware authentication.

Studies	Techniques	Classification Algorithms	# Users	Performance
[89]	File system, network access, GPS	NN, ED	8	90% accuracy, (28.8% (file-system), 46.4% (networked), 13.7% (combined)) FAR, (15.6% (file-system) 7% (networked) 11% (combined)) FRR
[90]	Online activities	LR	Users online history	about 85% accuracy, 0.03% EER
[91]	App-usage	HMMs	UMDAA-02, Securacy	16.16% EER
[92]	Bluetooth, WiFi	K-NN	200	85% accuracy, 13% EER
[93]	GPS	SVM	MDC Dataset	82.05% accuracy
[94]	GPS	W2V	CARS, SherLock	83% accuracy
[95]	GPS	Mot2vec	CDR	not given

**Table 10:** A list of continuous authentication surveys.

Topics	Year	Focus
Expanding continuous authentication with mobile devices [174]	2015	CA for IoT
Continuous and transparent multimodal authentication: Reviewing the state of the art [180]	2016	CA with multimodal biometrics
Continuous User Authentication on Mobile Devices: Recent Progress and Remaining Challenges [126]	2016	CA overview
Continuous Authentication and Authorization for the Internet of Things [181]	2017	CA for IoT
Who wants Continuous Authentication on Mobile Devices? [182]	2018	User opinion and experience
Leveraging user-related Internet of Things for continuous authentication: A survey [24]	2019	CA for IoT
A survey on different continuous authentication systems [175]	2019	CA overview
Data Behind Mobile Behavioural Biometrics—a Survey [178]	2020	Behavioral biometrics
Towards Wider Adoption of Continuous Authentication on Mobile Devices [177]	2020	Security and power consumption
Sensor-based Continuous Authentication of Smartphones’ Users Using Behavioral Biometrics: A Contemporary Survey [176]	2020	CA with behavioral biometrics
Touch-dynamics based Behavioural Biometrics on Mobile Devices—A Review from a Usability and Performance Perspective [183]	2020	Usability and Performance
Privacy-Preserving Sensor-Based Continuous Authentication and User Profiling: A Review [179]	2021	Privacy overview

## Data Availability

Not applicable.

## Abbreviations

The following abbreviations are used in this manuscript:

1DMRLBP	1-dimensional Multi-resolution Local Binary Patterns
AB	Ada Boost
BB	Behavioral Biometrics
BT	Bayesian Theory
CA	Continuous Authentication
CC	Cross-Correlation
CEW	Closed Eyes in the Wild
COTS	Commercial Off The Shelf
CNN	Convolutional Neural Network
CRR	Correct Rejection Rate
CRM	Cyclic Rotation Metric
DDM	Drift Detection Method
DTW	Dynamic Time Warping
ED	Euclidean Distance
ERR	Equal Error Rate
FAR	False Acceptance Rate
FC	Fuzzy Commitment
FF	Feed Forward
FFT	Fast Fourier Transform
FPR	False Positive Rate
FRR	False Rejection Rate
GAN	Genuine Authentication Rate
GDI	Gait Dynamics Images
HMM	Hidden Markov Model
HTER	Half Total Error Rate
Idp	Identity Provider
kNN	k-Nearest Neighbors
LBP	Local Binary Pattern
LDA	Linear Discriminative Analysis
LS	Least Square
Mot2vec	Motion-to Vector
LR	Linear Regression
MAD	Median Absolute Deviation
MHD	Modified Hausdorff Distance
MLP	Multi Layered Perception
NB	Naive Bayes
PCA	Principal Component Analysis
PSO-RBFN	Particle Swarm Optimization Radial Basis Function Network
RF	Random Forest
SP	Service Provider
SRC	Sparse Representation Classification
SVM	Support Vector Machine
TPR	True Positive Rate
W2V	Word2Vec
VAD	Voice Activity Detection
ZMCP	Ziv–Merhav Cross Parsing
